# Development and validation of a Brief Diet Quality Assessment Tool in the French-speaking adults from Quebec

**DOI:** 10.1186/s12966-019-0821-6

**Published:** 2019-08-06

**Authors:** J. Lafrenière, S. Harrison, D. Laurin, C. Brisson, D. Talbot, P. Couture, S. Lemieux, B. Lamarche

**Affiliations:** 1 Institute of Nutrition and Functional Foods, Laval University, Québec, Canada; 2 School of Nutrition, Laval University, Québec, Canada; 3 Population Health and Optimal Health Practices Research Unit, CHU de Québec-Laval University Research Center, Québec, Canada; 4 Faculty of Pharmacy, Laval University, Québec, Canada; 5 Department of Social and Preventive Medicine, Faculty of Medicine, Laval University, Québec, Canada; 6 Endocrinology and Nephrology unit, Centre de recherche du CHU de Québec, Laval University, Québec, Canada

**Keywords:** Diet quality, Classification and regression tree, Alternative healthy eating index, Brief diet quality assessment tool

## Abstract

**Background:**

The objective of this study was to develop and validate a short, self-administered questionnaire to assess diet quality in clinical settings, using the Alternative Healthy Eating Index (AHEI) as reference.

**Methods:**

A total of 1040 men and women (aged 44.6 ± 14.4 y) completed a validated web-based food frequency questionnaire (webFFQ) and had their height and weight measured (development sample). Participants were categorized arbitrarily according to diet quality (high: AHEI score ≥ 65/110, low: AHEI score < 65/110) based on dietary intake data from the webFFQ. The Brief Diet Quality Assessment Tool was developed using a classification and regression tree (CART) approach and individual answers to the webFFQ among participants considered to have a plausible energy intake (ratio of reported energy intake to basal metabolic rate ≥ 1.2 and < 2.4; *n* = 1040). A second sample of 3344 older adults (aged 66.5 ± 6.4 y) was used to test the external validity of the Brief Diet Quality Assessment Tool (external validation sample).

**Results:**

The decision tree included sequences of 3 to 6 binary questions, yielding 21 different pathways classifying diet quality as being high or low. In the development sample, the area under the receiver operating characteristic (ROC) curve of the predictive model was 0.92, with sensitivity, specificity and agreement values of 89.5, 83.9 and 87.2%. Compared with individuals having a low-quality diet according to the Brief Diet Quality Assessment Tool (mean AHEI 56.7 ± 11.4), individuals classified as having a high-quality diet (mean AHEI 71.3 ± 11.0) were significantly older, and had lower BMI, percent body fat and waist circumference, and had lower blood pressure, triglycerides, cholesterol/HDL ratio and fasting insulin as well as higher HDL-cholesterol concentrations (all *P* < 0.05). Similar results were observed in the external validation sample, although overall performance of the Brief Diet Quality Assessment Tool was slightly lower than in the development sample, with an area under the ROC curve of 0.79 and sensitivity, specificity and agreement values of 73.0, 69.0 and 71.3%, respectively.

**Conclusion:**

The CART approach yielded a simple and rapid Brief Diet Quality Assessment Tool that identifies individuals at risk of having a low-quality diet. Further studies are needed to test the performance of this tool in primary care settings.

**Electronic supplementary material:**

The online version of this article (10.1186/s12966-019-0821-6) contains supplementary material, which is available to authorized users.

## Background

One of the cornerstones of chronic disease prevention is to persuade the population to adhere to dietary guidelines [1]. For years, clinical guidelines have been largely focused on the concept of primary prevention, which aims to alleviate the impact of risk factors on chronic diseases. More recently, the notion of primordial prevention has emerged as a potentially more efficient public health strategy. Primordial prevention, for which optimizing diet is key, aims to avoid the development of risk factors in the first place [2]. However, physicians rarely inform their patients about the importance of healthy eating. In a Canadian study [3], family practitioners reported discussing diet with only 32% of their patients with type 2 diabetes and with less than 10% of their non-diabetic patients. One of the major challenges to implementing dietary counseling in a primary care setting is the lack of valid tools that assess diet quality, rapidly and accurately. In that regard, assessing global diet quality rather than relying on a few single nutrients of concern such as sodium and sugar is essential. A comprehensive approach to assessing diet quality, which takes into consideration food choices as well as interactions among foods and nutrients is more promising. Several complex dietary scores based on mathematical algorithms have been developed to describe the quality of the diet. The Alternative Healthy Eating Index (AHEI), which has been revised over the years to reflect current scientific literature, is well established [4]. It is based on extensive research on the association between foods and chronic disease risk [4, 5]. However, as with many other diet quality scoring systems [6–8], computing the AHEI score requires in-depth data collection and analyses of food and nutrient intakes, which is very difficult in clinical settings.

Food frequency questionnaires, which survey a list of foods and beverages consumed over a specific period, hence providing information on habitual food intake, are an important tool in nutrition research [9]. Although most of these questionnaires range from 80 to 120 questions and take up to 60 min to complete [10], shorter versions have been developed to assess diet quality [11–16]. Other short diet assessment tools have been developed to identify foods that contribute the most to the intake of specific nutrients such as saturated fat or sodium [13, 17]. Previously published data indicated that a diet quality score derived from such short questionnaires is weakly but significantly correlated with a diet quality score assessed using data from full dietary assessment questionnaires [18]. However, to the best of our knowledge, no Brief Diet Quality Assessment Tool has yet been developed specifically to predict a global diet quality score such as the AHEI.

The objective of this study was to develop and validate a short, simple and cost-effective Diet Quality Assessment Tool in French-speaking adults from the Province of Quebec, in Canada. The classification and regression tree (CART) approach was used for that purpose. We hypothesized that the CART approach yields a predictive model of diet quality that is simple and easy to use, and hence potentially transferable and useful in clinical settings.

## Methods

### Participants

**Fig. 1:**
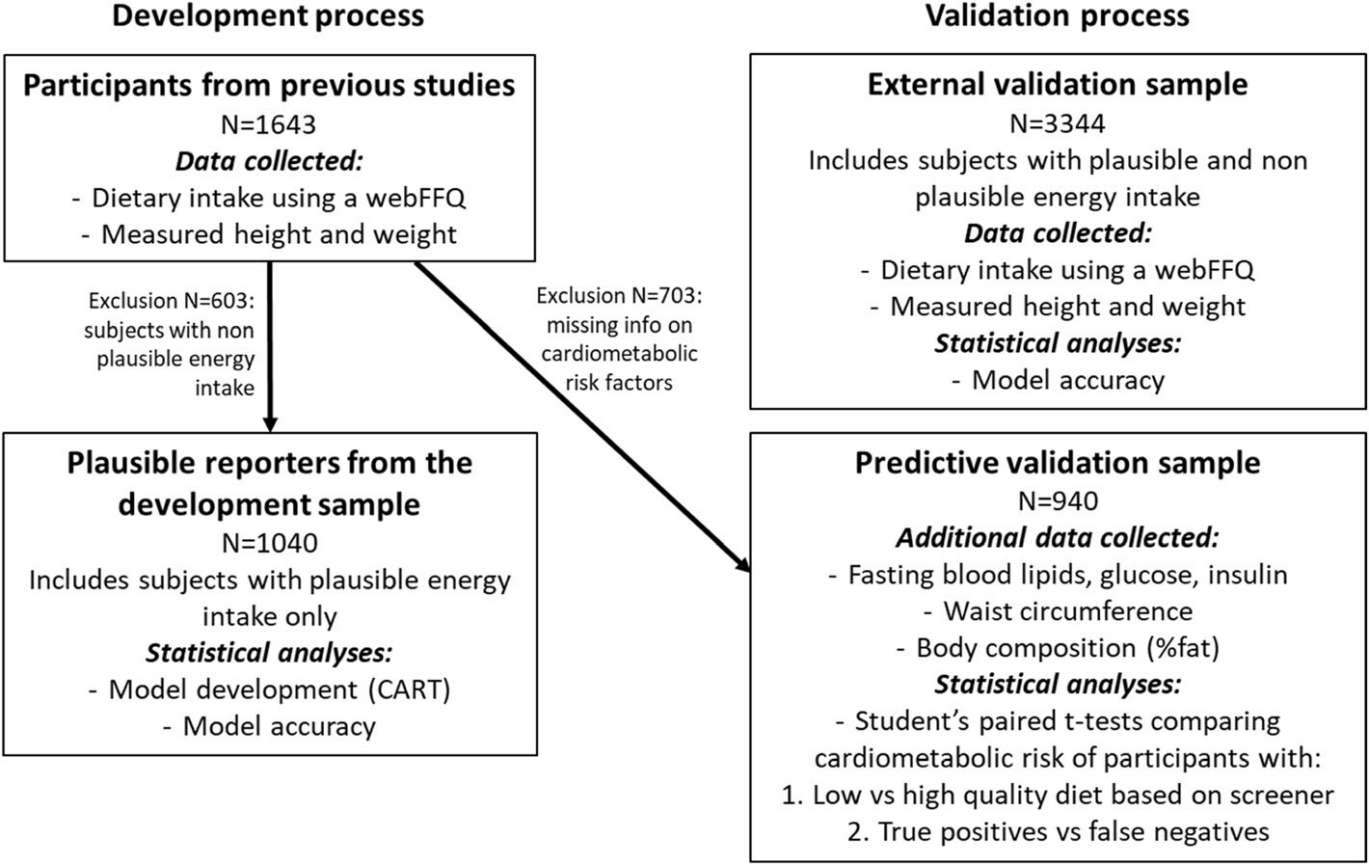
Flow diagram of the data collected, and statistical analyses performed within each sample and sub-sample for the development and the validation of the Brief Diet Quality Assessment Tool

This study is based on data from two main samples of participants, from which subsamples have been created for specific analyses, as detailed below. As shown in Fig. 1, the first sample (development sample) included 1643 healthy participants involved in 11 studies previously conducted at the Institute of Nutrition and Functional Food (INAF) over the years. All data were taken at the baseline of each study, prior to initiating any treatment or intervention, hence reflecting usual habits. The external validation sample comprised 3344 participants taking part in a longitudinal occupational study on cardiovascular health [19]. This external validation sample comprised older individuals, which is relevant to the brief assessment of diet quality since behavioural factors including diet are important predictors of morbidity and mortality in aging populations [20]. Moreover, individuals aged 65 and older are the most frequent users of primary care, therefore a key target population for rapid dietary assessment in such settings [21]. All participants lived in the Province of Quebec at the time of the study and spoke French as their primary language. All participants provided consent in written form to have their data included in a database for use in research other than the main project in which they participated. The protocol of each of these studies was in accordance with the declaration of Helsinki. Data used in this project are part of a data management framework approved by the Laval University Ethics Committee (2008–279 CG A-1 R-2).

### Assessment of cardiometabolic risk factors

Each participant visited the INAF or one of the affiliated research centers for at least one in-person data collection session. Height and weight were measured by trained staff. A sub-sample of 940 individuals from the development sample, referred to as the predictive validation sample, provided a 12-h fasting blood sample and had their blood pressure, body composition and waist circumference measured. Blood samples were immediately centrifuged at 17 °C for 10 min at 1100×g to obtain serum samples, which were stored at − 80 °C until processed. Serum total cholesterol, triglycerides, and HDL-cholesterol concentrations were assessed with the use of a Roche Modular P system (Roche Diagnostics, Mannheim, Germany). LDL-cholesterol was calculated using the Friedewald equation [22]. Fasting blood glucose concentrations were measured by colorimetry (Hexokinase Method, Roche Modular P System), whereas insulin concentrations were measured with the use of electrochemiluminescence (Cobas 6000, Roche Diagnostics). Systolic and diastolic blood pressures were determined from the means of 3 consecutive measurements that were taken 3 min apart in a sitting position after a 10-min rest with the use of an automated blood pressure monitor (Digital BPM HEM-907XL model; Omron). Percent body fat was determined by the body composition analyzer BC-418 (Tanita, Arlington Heights, II). Waist circumference measurements were taken at the end of a normal expiration with a tape placed horizontally directly on the skin at mid-distance between the last rib and the top of the iliac crest. Waist circumference was determined as the mean of three measurements at the nearest 0.1 cm.

### Dietary assessment

All participants from the development sample and the external validation sample completed the same validated web-based food frequency questionnaire (webFFQ) [23] at home, from which the AHEI was calculated as proposed by Chuive et al. [4]. The scoring method is presented in Table 1. All questions from the webFFQ are structured similarly. Frequency of consumption is first assessed based on up to 8 predetermined answers. Participants then provide information on portion size using up to 6 image options. This sequence is cognitively easier for respondents [24].

**Table 1:** Alternative Healthy Eating Index (AHEI)-2010 scoring method

Components	Criteria for minimum score (0)	Criteria for maximum score (10)
Vegetables, servings/day	0	≥5
Fruit, servings/day	0	≥4
Whole grains, g/day	0	
Women		75 (approximately 5 servings/day)
Men		90 (approximately 6 servings/day)
Sugar-sweetened beverages and fruit juice, servings/day	≥1	0
Nuts and legumes, servings/day	0	≥1
Red/processed meat, servings/day	≥1.5	0
Trans fat, % energy	≥4	≤0.5
Long chain (n-3) fats (EPA + DHA), mg/day	0	250
PUFA, % energy	≤2	≥10
Sodium, mg/day	Highest decile	Lowest decile
Alcohol, drinks/day^a^		
Women	≥2.5	0.5–1.5
Men	≥3.5	0.5–2.0
Total	0	110

^a^In the design of the AHEI, authors assigned the highest score to moderate, and the lowest score to heavy, alcohol consumers. The non-drinkers received a score of 2.5. One drink is 4 oz. of wine, 12 oz. of beer, or 1.5 oz. of liquor (1 oz. = 28.35 g)

Adapted from Chiuve et al. (2012) [4]

Among participants in the development sample, self-reported energy intake (rEI) was estimated using dietary intake data derived from the webFFQ and estimated basal metabolic rate (eBMR) was calculated using the Mifflin-St Jeor equation [25]. We considered, based on the Goldberg cut off [26], that participants with a ratio of rEI:eBMR ranging from 1.2 to 2.4 were plausible reporters. Data from non-plausible reporters based on these criteria in the development sample were excluded from the model development analysis because using potentially invalid data from individuals with over or under-reporting food intake to develop the Brief Diet Quality Assessment Tool may have yielded spurious associations between food intake and diet quality. However, all plausible and non-plausible reporters were included in the external validation sample in order to test the validity of the Brief Diet Quality Assessment Tool in a context that more closely reflects real life conditions, where the risk of over or underreporting is not assessed and therefore unknown.

### Development of the brief diet quality assessment tool

The CART approach was used to develop the Brief Diet Quality Assessment Tool in the development sample. CART is a statistical approach of supervised learning that draws food patterns and identifies best predictors of an outcome among a list of variables [4]. This type of algorithm is used to split a sample of independent variables in mutually exclusive subgroups based on common traits [20]. By design, the tool identifies individuals at risk of having a diet of low quality, so that they may receive adequate guidance. By default, all remaining individuals who do not fall into this category have a high probability of having adequate dietary habits. The AHEI was considered the outcome variable, while answers to individual questions in the webFFQ as well as food groups were used as predictors. Overall diet quality was arbitrarily categorized as high (AHEI ≥65/110) or low (AHEI< 65/110) to develop the Brief Diet Quality Assessment Tool. This cut-off was chosen based on the observation that individuals with a score of 65/110 and above are at a lower risk of major chronic disease compared with those with a lower score [4]. Information from the webFFQ was converted into equivalent of servings per day for the analysis using standard references in Canada. Of the 136 questions of the webFFQ, 117 were included in the analysis.

As the webFFQ measures food intake with a high degree of specificity for some foods, it was decided to exclude right from the beginning questions that were considered too specific or irrelevant for use in a Brief Diet Quality Assessment Tool. For example, questions from the webFFQ that did not specifically indicate the type of foods consumed (e.g. “How often do you eat other types of bread?”) were not considered in the developing the CART. A total of 27 categories were created to generate meaningful food groups based on the categorization proposed in Canada’s Food Guide [27] as well as through consensus within the research team. Specifically, a subgroup was created for the different forms of cow milk (low, regular fat), and for all types of milk (including plant-based milks), of yogurt, and of cheese. Subgroups were also created for processed meats (including cold cuts, nuggets, bacon, terrines and sausages) and the different types of fish, breads, cereals, rice, pasta, chocolate and peanut butter were also grouped each in single food categories. Other subgroups were created for processed foods such as muffins, pancakes, pizza, sub sandwiches, cookies, cakes, pies, as well as for soft drinks, tea and coffee, desserts as well as nutritional supplements. Finally, food subgroups reflecting added sugar (in tea or coffee) and added fat were also created. Although most of the questions from the webFFQ are related to specific food items, some refer to a series of foods that have similar nutritional composition (e.g. “How often do you eat broccoli, green and yellow beans, Brussels sprouts, turnips, beets, asparagus, cabbage, mushrooms and mixed vegetables”).

Age and sex, which are known to influence diet quality, were also considered as covariates in the models [28, 29]. The complete list of variables used to develop the Brief Diet Quality Assessment Tool is available in the Additional file 1. Overfitting was controlled using tenfold Monte Carlo cross-validation [30]. The CART modeling was performed using the statistical program R and the package Rpart with version 3.3.2 (R Foundation for Statistical Computing, Vienna, Austria).

### Statistical analysis

In plausible reporters from the development sample, accuracy of the Brief Diet Quality Assessment Tool was assessed by calculating sensitivity (the probability of being classified by the tool as having a diet of low quality among those with an AHEI < 65/110), specificity (the probability of being classified by the tool as having a diet of high quality among those with an AHEI ≥ 65/110), agreement (proportion of respondents adequately categorized by the tool), positive predictive value (PPV; the probability of an AHEI < 65/110 in those classified by the tool as having a diet of low quality), negative predictive value (NPV; the probability of an AHEI ≥ 65/100 in those classified by the tool as having a diet of low quality) and the area under the Receiving Operating Characteristic (ROC) curve.

### Predictive validation

In the predictive validation sample, Student’s paired t-tests were used to compare the cardiometabolic risk profile of participants classified by the Brief Diet Quality Assessment Tool as having a low or high-quality diet and between true positives (individuals with an AHEI < 65 correctly classified as having a low-quality diet) and false negatives (individuals with an AHEI < 65 incorrectly classified as having a high-quality diet).

### External validation

External validation of the Brief Diet Quality Assessment Tool was undertaken using data from the external validation sample and the accuracy metrics described above (ie. sensitivity, specificity, agreement, PPV, NPV and area under the ROC curve) with the AHEI as reference.

XLSTAT 2017 (Addinsoft, Paris, France) was used to assess accuracy of the Brief Diet Quality Assessment Tool while SAS version 9.4 (SAS Institute Inc., NC, USA) was used for all other statistical analyses.

## Results

Characteristics of all plausible reporters from the development sample (*N* = 1040) and all participants from the external validation sample (*N* = 3344) are presented in Table 2. Participants in the external validation sample were older, had slightly but significantly lower body mass index (BMI, *P* = 0.01) and higher AHEI score (*P* < 0.001) compared with those in the development sample.

**Table 2:** Participants’ characteristics in plausible reporters from the development sample and external validation samples

	Plausible reporters from the development sample (*n* = 1040)			External validation sample (*n* = 3344)		
	All participants (*n* = 1040)	Women (*n* = 536)	Men (*n* = 504)	All participants (*n* = 3344)	Women (*n* = 1614)	Men (*n* = 1730)
AHEI score	61.9 (14.1) [18.9–99.0]	65.2 (13.2) [29.2–99.0]	58.4 (14.3) [18.9–91.4]	63.4 (12.9) [22.9–100.8]	65.8 (12.6) [26.6–100.8]	61.3 (12.8) [22.9–99.8]
Age (years)	45.4 (14.2) [18.0–72.0]	45.3 (14.4) [18.0–70.0]	45.5 (14.0) [18.0–72.0]	66.5 (6.4) [47.0–91.0]	64.8 (5.9) [47.0–87.0]	68.2 (6.4) [47.0–91.0]
BMI (kg/m^2^)	27.7 (5.3) [16.3–54.7]	26.9 (5.5) [16.3–54.7]	25.5 (5.0) [17.2–48.2]	27.1 (4.8) [13.6–56.1]	26.7 (5.4) [13.6–56.1]	27.5 (4.2) [14.7–48.0]

Values are presented as mean (standard deviation) and range [minimum-maximum]

*AHEI* Alternate Healthy Eating Index, *BMI* Body mass index

**Fig. 2:**
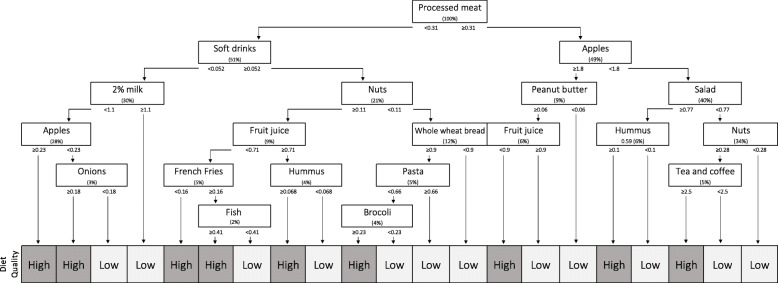
Classification and regression tree for the prediction of diet quality (*N* = 1040). Each box represents a split of the sample according to a specific food item predicting the diet quality outcome. The percentage of the total sample used at each split is shown in parentheses. Cut-offs of binary division are selected by the model to create the two most distinctive subgroups based on the diet quality outcome to predict (low vs. high). Cut-offs in servings/day are presented for each split under the box. Each sequence of questions yields the predicted diet quality (low or high)

Figure 2 presents the output of the decision tree produced by the CART in the plausible reporters from the development sample. Each split represents the question of the webFFQ that best differentiates the dietary outcome (i.e. diet of low vs. high quality). The cut-offs, expressed in servings per day, are determined by the model itself. The exact same model with the same cut-offs were used for the validation process. Color coded terminal leaves classify the respondents as having a low or a high-quality diet according to the AHEI cut-off of 65/110. Most of the 16 variables included in the final model are directly related to individual components of the AHEI. The first split corresponds to the intake of processed meat, which comprised questions on cold cuts, nuggets, bacon, terrines and sausages. Questions related to the intake of vegetables (broccoli, onion and salad), fruit (apples, which referred to the question: “how often are you eating apples, tangerines, oranges, pears, nectarines or peaches?”), whole grains (whole-grain bread), sugar-sweetened beverages and fruit juice (soft drinks and fruit juice), nuts and legumes (nuts, hummus and peanut butter), long chain (n-3) fatty acids (fish), sodium (French fries and processed meat) are also integral part of the AHEI calculation. Three questions that yielded a decisive split in the CART model were not directly associated with components of the AHEI, namely, 2% M.F. milk, pasta and the grouping of tea and coffee.

Accuracy of the Brief Diet Quality Assessment Tool to identify individuals with a low diet quality (AHEI < 65) in the development sample is presented in Table 3. The Brief Diet Quality Assessment Tool had a high area under the ROC curve (0.92) and PPV (0.90). Other metrics were generally consistent with high accuracy (range 0.84–0.88). Comparative analysis of the cardiometabolic risk profile of individuals with a predicted low and high diet quality in the predictive validation sample is shown in Table 4. As expected, both men and women classified by the Brief Diet Quality Assessment Tool as having a low-quality diet had a significantly lower AHEI score than those classified as having a high-quality diet. Furthermore, individuals classified as having a low-quality diet by the Brief Diet Quality Assessment Tool were younger, had a higher BMI and waist circumference and, globally, a deteriorated cardiometabolic profile compared to those classified as having a high-quality diet. False negatives (individuals with an AHEI < 65 incorrectly classified as having a high-quality diet by the Brief Diet Quality Assessment Tool) had a higher AHEI score and a more favourable cardiometabolic profile than true positives (individuals with an AHEI < 65 correctly classified as having a low-quality diet, Table 5). Finally, Table 6 presents accuracy metrics of the Brief Diet Quality Assessment Tool in the external validation sample. All metrics were significantly lower in the external validation sample than in the development sample (sensitivity, specificity and agreement values of 73.0, 69.0 and 71.3%, respectively).

**Table 3:** Accuracy of the Brief Diet Quality Assessment Tool to predict low diet quality in the plausible reporters from the development sample (*N* = 1040)

	Accuracy metrics (95% CI)
Sensitivity	0.88 (0.86–0.91)
Specificity	0.85 (0.86–0.88)
Agreement	0.87 (0.85–0.89)
Positive predictive value	0.90 (0.87–0.92)
Negative predictive value	0.84 (0.80–0.87)
AUC of the ROC	0.92 (0.90–0.94)

*AUC of the ROC* Area under the receiver operating characteristic curve

**Table 4:** Cardiometabolic risk profile by categories of diet quality predicted by the Brief Diet Quality Assessment Tool in the predictive validation sample (*N* = 940)^d^

	Diet quality according to Brief Diet Quality Assessment Tool					
	All		Women		Men	
	High (*N* = 373)	Low (*N* = 567)	High (*N* = 227)	Low (*N* = 251)	High (*N* = 146)	Low (*N* = 316)
AHEI score (/110)	71.5 (11.4)	53.1 (11.2)^c^	72.3 (11.5)	55.2 (10.2)^c^	70.2 (11.1)	51.4 (11.6)^c^
Age (years)	46.2 (13.5)	42.5 (13.1)^c^	45.9 (13.4)	41.7 (13.2)^c^	46.5 (13.5)	43.1 (13.0)^b^
BMI (kg/m^2^)	25.9 (5.2)	28.6 (6.5)^c^	25.3 (5.2)	28.9 (7.3)^c^	26.8 (5.1)	28.5 (5.8)^b^
Waist circumference (cm)	87.3 (14.6)	96.0 (16.6)^c^	83.0 (13.1)	92.1 (16.7)^c^	93.9 (14.4)	99.1 (15.8)^c^
Percent fat (%)	28.9 (9.1)	30.2 (10.4)^a^	33.0 (7.5)	37.3 (8.7)^c^	22.6 (7.6)	24.6 (7.9)^b^
*Blood pressure (mmHg)*						
Systolic	114.9 (14.6)	119.9 (13.6)^c^	110.8 (14.2)	115.2 (13.8)^c^	121.3 (12.6)	123.6 (12.3)
Diastolic	71.3 (9.9)	75.3 (10.1)^c^	69.8 (10.0)	74.1 (10.1)^c^	73.6 (9.3)	76.3 (10.0)^b^
*Lipids (mmol/l)*						
TG	1.2 (0.7)	1.5 (1.1)^c^	1.2 (0.7)	1.4 (1.0)^a^	1.2 (0.7)	1.5 (1.1)^c^
LDL-cholesterol	2.8 (0.9)	2.9 (0.9)	2.8 (1.0)	2.9 (0.9)	2.8 (0.8)	2.9 (0.9)
HDL-cholesterol	1.5 (0.5)	1.4 (0.4)^c^	1.6 (0.5)	1.5 (0.4)^c^	1.4 (0.4)	1.3 (0.4)^b^
Chol/HDL-Cholesterol	3.4 (1.1)	3.8 (1.4)^c^	3.3 (1.2)	3.7 (1.4)^b^	3.6 (1.1)	4.0 (1.3)^b^
Glucose (mmol/L)	5.1 (0.7)	5.3 (1.0)^a^	5.1 (0.7)	5.1 (0.8)	5.3 (0.7)	5.4 (1.3)
Insulin (pmol/L)	88.1 (49.8)	107.0 (77.6)^c^	86.1 (54.6)	103.6 (60.4)^c^	91.3 (41.0)	109.7 (88.9)^b^

Variables are presented as mean (standard deviation)

*AHEI* Alternate Healthy Eating Index, *BMI* Body mass index

^a^*P* < 0.05, ^b^*P* < 0.01, ^c^*P* < 0.001, from the Student’s t-test for the difference between participants classified as having a high diet quality or a low diet quality

^d^Only men and women from the development sample among whom cardiometabolic risk factors were measured were included in this analysis. This is why the number of participants (total *N* = 940) is lower than in the entire development sample (*N* = 1643)

**Table 5:** Cardiometabolic risk profile of participants with an AHEI < 65/110 from the predictive validation sample, classified as true positive and false negative based on the Brief Diet Quality Assessment Tool (*N* = 592)^a^

	All participants with AHEI < 65		
	True positives (*N* = 500)	False negative (*N* = 92)	*P* ^1^
AHEI score (/110)	50.7 (9.4)	56.3 (7.0)	< 0.001
Age (years)	42.0 (13.2)	42.5 (12.9)	0.77
BMI (kg/m^2^)	28.8 (6.6)	26.6 (5.2)	< 0.001
Waist circumference (cm)	96.4 (17.0)	90.0 (14.4)	< 0.001
Percent fat (%)	30.2 (10.4)	29.6 (9.0)	0.60
*Blood pressure (mmHg)*			
Systolic	120.1 (13.7)	115.5 (13.5)	0.003
Diastolic	75.6 (10.1)	72.6 (9.1)	0.01
*Lipids (mmol/l)*			
TG	1.5 (1.1)	1.2 (0.7)	< 0.001
LDL-cholesterol	2.9 (0.9)	2.8 (0.8)	0.42
HDL-cholesterol	1.4 (0.4)	1.5 (0.5)	0.06
Chol/HDL-cholesterol	3.9 (1.4)	3.5 (1.1)	0.006
Glucose (mmol/L)	5.3 (1.2)	5.2 (0.8)	0.42
Insulin (pmol/L)	109.2 (80.3)	91.0 (42.8)	0.002

Variables are presented as mean (standard deviation)

*AHEI* Alternate Healthy Eating Index, *BMI* Body mass index

^1^*P* value of the Student’s t-test for the difference between participants classified true positive (AHEI< 65 and classification as low diet quality with the Brief Diet Quality Assessment Tool) and false negative (AHEI< 65 and classification as high diet quality with the Brief Diet Quality Assessment Tool)

^a^This analysis included only individuals from the predictive validation sample among whom the AHEI was < 65 (total *N* = 592)

**Table 6:** Accuracy of the Brief Diet Quality Assessment Tool to predict low diet quality in the external validation sample (*N* = 3344)

	Accuracy metrics (95% CI)
Sensitivity	0.73 (0.71–0.75)
Specificity	0.69 (0.67–0.71)
Agreement	0.71 (0.70–0.73)
Positive predictive value	0.77 (0.75–0.79)
Negative predictive value	0.64 (0.62–0.67)
AUC of the ROC	0.79 (0.77–0.80)

*AUC of the ROC* Area under the receiver operating characteristic curve

## Discussion

The objective of this study was to develop and validate a short and simple questionnaire to assess diet quality for potential use in a clinical and primary care setting. Using the CART modeling approach, the analysis yielded a Brief Diet Quality Assessment Tool that comprises a maximum of six questions, with acceptable accuracy metrics to identify individuals likely to have a diet of low quality. Predictive validation of the Brief Diet Quality Assessment Tool using cardiometabolic risk factors provided further evidence of adequate performance to identify individuals at risk of having a low diet quality. External validation analyses in a sample of older adults also showed relatively good predictive values, although the model was overall less accurate than in the sample in which it was developed. Therefore, this suggests that Brief Diet Quality Assessment Tool has interesting potential for use in a primary care setting, as it identifies individuals at risk of having a low-quality diet and hence with the greatest needs in terms of nutritional support and guidance.

We are unaware of other studies where brief assessment tools of global diet quality have been developed using detailed dietary assessment methods such as the AHEI as reference. Cook et al. [31] have developed three questionnaires of one and five questions to predict fruit and vegetable consumption. Although more than 80% of high fruit consumers were correctly identified by the single question questionnaire, only 56% of the individual identified as high fruit consumers were true positives. For vegetables, the sensitivity of different options of the model ranged from 36 to 70% and the PPV from 26 to 39%. Similarly, Teal et al. [14] created a brief assessment tool for excessive fat consumption that could reasonably identify high fat consumers (PPV of 81%) but not those with a lower fat intake (NPV of 39%). Dietary assessment tools developed with supervised learning approaches appear to yield higher accuracy metrics. Indeed, using stepwise multiple logistic regression, Glümer et al. [32] developed and validated a screening tool for type 2 diabetes in the Danish population that demonstrated good sensitivity (73%) and specificity (74%). Using the CART approach, Xie et al. [33] developed a diabetes screening tool that was more sensitive and specific in women than in men (61% vs. 59 and 71% vs. 63%, respectively). In the present study, the Brief Diet Quality Assessment Tool presented adequate accuracy metrics. Sensitivity was high with 88% of individuals with AHEI < 65 adequately correctly classified as having a low-quality diet. Specificity, reflecting the capacity of the Brief Diet Quality Assessment Tool to correctly identify individuals not at risk of having a poor diet, was also high at 85%. The area under the ROC curve was 0.92, indicating that the AHEI cut-off of 65/110 was optimal to generate a maximal proportion of true positives over false positives.

A higher AHEI score has been associated with higher HDL-cholesterol concentrations [34] and lower waist circumference [35], blood pressure and triglyceride levels [36] in different populations. Our data are consistent with these observations by showing significant differences in cardiometabolic risk between participant categorized with the Brief Diet Quality Assessment Tool as having a high or a low-quality diet for almost all variables tested. Even if the Brief Diet Quality Assessment Tool did not correctly classify all participants, individuals with an AHEI < 65 who were misclassified as having a high-quality diet (false negative) had a more favorable cardiometabolic risk profile when compared with true positive individuals. Indeed, in addition to presenting a higher AHEI, false negatives had lower BMI, waist circumference, blood pressure, serum TG, insulin and cholesterol/HDL-cholesterol ratio compared with true positives. This observation alleviates the consequences of misclassifying someone with a low diet quality.

In the external validation sample, accuracy metrics of the Brief Diet Quality Assessment Tool were lower than in the development sample. This was anticipated as the CART algorithm was specifically built based on data from the development sample. However, the Brief Diet Quality Assessment Tool performed reasonably well in this independent sample with sensitivity and specificity values of 73 and 69%, respectively. Other investigators have also observed lower metrics of accuracy of the predictive model when testing its external validity [32].

The accuracy metrics yielded by the Brief Diet Quality Assessment Tool needs to be contextualized for its potential use in a clinical primary care setting and according to the consequences of false positive or negative classifications. In a clinical setting, individuals classified as having low-quality diet based on the Brief Diet Quality Assessment Tool may be offered to meet with a dietician, who will inevitably assess dietary habits using more comprehensive dietary assessment methods and confirm their status. False positives, i.e. those presumably at risk of having a low-quality diet but who in fact have an AHEI ≥65, would unnecessarily use dietary counseling resources until their true dietary status is confirmed by more comprehensive assessment methods. Meanwhile, false negatives, i.e. individuals incorrectly classified as having a high-quality diet, will most likely maintain suboptimal dietary habits until further assessment. However, our data indicated that false negative individuals had a less deteriorated cardiometabolic risk profile than true positive individuals. This suggests a higher degree of “tolerance” before actions can be formally implemented to address the issues of diet quality in these patients. Finally, health practitioners need to acknowledge that this Brief Diet Quality Assessment Tool is not intended to be a precise dietary assessment tool. The primary function of this new tool is to bring the topic of diet quality to the discussion and potentially awaken consciences of both patients and physicians about this key aspect of preventive medicine.

### Strengths and limitations

The use of the CART approach in this study is highly original and can be considered an important strength. This type of algorithm is used to split a sample of independent variables in mutually exclusive subgroups based on common traits [37]. The end product is visually meaningful and can be easily interpreted by non-statisticians [38]. Other supervised learning methods and regression analysis have been used in the past with slightly better accuracy, but their translation into visually attractive tools is challenging [39, 40]. Each CART is also inherently representative of the population in which it was developed. This ensures the generalizability at a local level, which is not guaranteed with tools validated elsewhere [41]. Consequently, this approach has a limited reproducibility in populations other than French-speaking adults of Quebec, in which food habits could be different. The CART approach has also been shown to be prone to classification errors [39]. Such errors apparently did not materially affect the performance of the model predicting diet quality in the development sample. The main advantage of this supervised learning strategy is to maximize specificity while limiting the number of questions by grouping the respondents in subgroups. Indeed, unlike calculating the AHEI using detailed and comprehensive dietary assessment methods, the Brief Diet Quality Assessment Tool can be self-administered within few minutes and interpreted without diet analyzing software. As indicated above, brief dietary assessment tools cannot substitute more comprehensive methods when detailed results are needed for counseling. Finally, even if the final model may be deceptive to some because of the small number of questions it comprises, it is important to highlight that the CART approach identified, from a broad series of foods, those that most closely predict an objective diet quality score, the AHEI, while ignoring other foods that did not statistically contribute to predicting this outcome [42].

There are limitations associated with the use of an FFQ to assess dietary intake, including a certain degree of misreporting [43], despite thorough validation [23]. However, the AHEI has been developed using dietary intake data from FFQs [4]. The Brief Diet Quality Assessment Tool was developed in a sample that excluded individuals with non plausible energy intake. Meanwhile, external validation was undertaken in a sample of individuals that did not exclude non plausible reports. The external validation sample in the present study was composed of older adults than individuals included in the development sample. While this may have attenuated the external validity of the tool to identify those with a low-quality diet, this approach more closely reflects real-life contexts, in which reporting status is unknown when assessing diet quality. We also acknowledge that the development sample may be biased as it includes participants involved in previous nutrition-related studies who might have a pre-existing interest in nutrition, thereby potentially influencing food choices and behaviors. Nevertheless, our analysis demonstrated that the Brief Diet Quality Assessment Tool may be useful in groups of adults with various characteristics.

## Conclusion

We have developed and validated an easy-to-use Brief Diet Quality Assessment Tool that classifies individuals according to their risk of having a diet of low quality. Individuals classified as having a diet of low quality had a deteriorated cardiometabolic risk profile compared with those classified as having a diet of better quality, a strong predictive validation demonstration. This Brief Diet Quality Assessment Tool could easily be implemented in a primary care setting, where dietary assessment is highly challenging due to limited resources and expertise. Future research includes extensive testing of a web-based version of the Brief Diet Quality Assessment Tool with different health professionals and populations in primary care settings. Testing the external validity of the tool in other populations is also imperative before it can be recommended for use.

## Additional file

Additional file 1: Complete list of variables potentially predictive of diet quality. (DOCX 19 kb)

## Data Availability

The datasets used and/or analyzed during the current study are available from the corresponding author on a reasonable request.

## Abbreviations

AHEI: Alternative healthy eating index
CART: Classification and regression tree
INAF: Institute of nutrition and functional food
NPV: Negative predictive value
PPV: Positive predictive value
ROC: Receiving Operating Characteristic
webFFQ: Web-based food frequency questionnaire
