# Supplementary material for: Development and validation of a Brief Diet Quality Assessment Tool in the French-speaking adults from Quebec
Source: Int J Behav Nutr Phys Act. 2019 Aug 6;16:61. doi: 10.1186/s12966-019-0821-6 (PMC6685233; doi:10.1186/s12966-019-0821-6)
Supplement: Supplementary file 1 — Complete list of variables potentially predictive of diet quality. (DOCX 19 kb) [file 12966_2019_821_MOESM1_ESM.docx]

**Additional file 1**: Complete list of variables potentially predictive of diet quality

| Age |
| --- |
| Sex |
| Apple (Apples/ tangerines/ oranges/ pears/ nectarines/ peaches/ bananas) |
| Avocado |
| Bacon |
| Bagel whole wheat |
| Bagel white |
| Barley (Barley/ bulgur/ couscous/ quinoa/ millet/ buckwheat) |
| Beer light |
| Beer no alcohol |
| Beer regular |
| Berries (Strawberries/ raspberries/ blueberries/ blackberries/ cranberries) |
| Broccoli (Green or yellow beans/ broccoli/ Brussel sprouts/ mixed vegetables/ turnip/ beetroot/ asparagus/ cabbage/ mushrooms) |
| Candies |
| Canned fruits |
| Cantaloupe (Cantaloupe/ honeydew melon/ watermelon |
| Carrot (Celery/ peppers/ carrots/ cucumbers/ dill pickles) |
| Cereal bars |
| Cherries (Fresh grapes/ cherries) |
| Chicken |
| Chicken skin |
| Chicken wings |
| Chips |
| Coffee |
| Compote fruit |
| Cordial |
| Corn |
| Cottage cheese (ricotta) |
| Crackers |
| Cream 35% |
| Cream soup |
| Croissant (Croissant/ vol-au-vent) |
| Doughnut |
| Dressing |
| Dried fruit (Raisins/ dried cranberries/ dates/ figs/ prunes) |
| Eggroll |
| Egg |
| Energy drink |
| Flavored hot drink |
| French baguette |
| French Fries |
| Fruit juice |
| Frozen yogurt (Frozen yogurt/ sorbet/ ice milk/ non-fat ice cream) |
| Grapefruit |
| Ground meat |
| Hamburger |
| Hamburger vegetarian |
| Hamburger chicken |
| Hotdogs |
| Hotdog vegetarian |
| Hummus |
| Ice cream (Ice cream/ fudge bars/ ice cream bars/ sundae) |
| Jam (Fruit jams/ jellies/ Nutella/ maple syrup/ maple butter/ honey/ chocolate syrup) |
| Kiwi (Clementines/ plums/ apricots/ kiwis) |
| Legume in tomato sauce |
| Liquor |
| Lobster (Lobster/ crab) |
| Mango (Mango/ pineapple) |
| Milk 0% M.F. |
| Milk 1% M.F. |
| Milk 2% M.F. |
| Milk 3.25% M.F. |
| Milk 3.9% M.F. |
| Desserts containing cow milk, soy milk or silken tofu |
| Meal replacement |
| Meat pie |
| Mussels (Mussels/ oysters) |
| Non-carbonated fruit drinks |
| Nuggets |
| Nuts |
| Olive |
| Onions |
| Organ |
| Parmesan fondue |
| Pesto |
| Pita (Pita bread/ tortilla) |
| Philadelphia |
| Popcorn |
| Potato |
| Poutine |
| Pretzel |
| Processed meat |
| Quiche |
| Red meat (Beef/ veal/ pork/ lamb/ game/ horse/ other red meat) |
| Red meat visible fat |
| Regular sour cream |
| Salad |
| Salmon pie |
| Salt added |
| Sauce meat |
| Sausages |
| Seafood |
| Soya beverage |
| Sport drink |
| Squash (Dark orange (winter) or summer squash/ marrow/ zucchini/ eggplant) |
| Sugar added |
| Sushi |
| Sweetener added |
| Tea decaffeinated |
| Tea regular |
| Terrine (Liver pate/ terrine/ cretons/ head cheese) |
| Tofu (Tofu/ tofu spread/ seitan/ soy based products) |
| Tomatoes |
| Tomato sauce |
| Tomato sauce with legumes |
| Tomato sauce with meat |
| Tomato sauce with tofu |
| Vegetable or tomatoes juice |
| Vegetable soup |
| Velveeta |
| Waffles (Waffles/ French toasts) |
| Water (Tap water/ bottled water/ mineral water/ herbal tea/ soda water) |
| Whipped cream topping |
| White bread |
| White sauce |
| Whole bread |
| Wine red |
| Wine rose |
| Wine white |
| All cow’s milk (grouping) |
| All milks (grouping) |
| All yogurts (grouping) |
| All cheeses (grouping) |
| All processed meats (grouping) |
| All fish (grouping) |
| All breakfast cereals (grouping) |
| All breads (grouping) |
| All muffins (grouping) |
| All pancakes (grouping) |
| All rice (grouping) |
| All types of tea and coffee (grouping) |
| All pizzas (grouping) |
| All sub sandwiches (grouping) |
| All chocolates (grouping) |
| All cookies (grouping) |
| All cakes (grouping) |
| All pies (grouping) |
| All desserts (grouping) |
| All types of peanut butter (grouping) |
| All types of soft drinks (grouping) |
| All types of pastas (grouping) |
| All dietary supplements (grouping) |
| Fat added to bread (grouping) |
| Fat added to vegetables (grouping) |
| Fat added to meat (grouping) |
| Sugar added to tea or coffee (grouping) |
